# Cancers Attributable to Overweight and Obesity From 2012 to 2014 in Nigeria: A Population-Based Cancer Registry Study

**DOI:** 10.3389/fonc.2019.00460

**Published:** 2019-06-11

**Authors:** Michael K. Odutola, Temitope Olukomogbon, Festus Igbinoba, Theresa I. Otu, Emmanuel Ezeome, Ramatu Hassan, Elima Jedy-Agba, Sally N. Adebamowo

**Affiliations:** ^1^Office of Strategic Information and Research, Institute of Human Virology, Abuja, Nigeria; ^2^Department of Oncology, National Hospital, Abuja, Nigeria; ^3^Department of Hematology, University of Abuja Teaching Hospital, Gwagwalada, Nigeria; ^4^Department of Surgery, University of Nigeria Teaching Hospital Enugu, Enugu, Nigeria; ^5^Federal Ministry of Health, Abuja, Nigeria; ^6^International Research Center of Excellence, Institute of Human Virology, Abuja, Nigeria; ^7^Division of Cancer Epidemiology, Department of Epidemiology and Public Health, University of Maryland School of Medicine, Baltimore, MD, United States; ^8^University of Maryland Marlene and Stewart Greenebaum Comprehensive Cancer Center, University of Maryland School of Medicine, Baltimore, MD, United States; ^9^Department of Research, Center for Research and Bioethics, Ibadan, Nigeria

**Keywords:** overweight, obesity, cancer, incidence, cancer registry, Nigeria

## Abstract

**Background:** Overweight and obesity are known risk factors for chronic diseases including cancers. In this study, we evaluated the age standardized incidence rates (ASR) and proportion of cancers attributable to overweight and obesity in Nigeria.

**Methods:** We obtained incidence data from the databases of two population-based cancer registries (PBCRs) in Nigeria (Abuja and Enugu cancer registries), on cancer site for which there is established evidence of an association with overweight or obesity based on the International Agency for Research on Cancer (IARC) and the World Cancer Research Fund (WCRF) classification. We analyzed the data using population attributable fraction (PAF) for overweight or obesity associated cancers calculated using prevalence data and relative risk estimates in previous studies.

**Results:** The two PBCRs reported 4,336 new cancer cases (ASR 113.9 per 100,000) from 2012 to 2014. Some 21% of these cancers were associated with overweight and obesity. The ASR for overweight and obesity associated cancers was 24.5 per 100,000; 40.7 per 100,000 in women and 8.2 per 100,000 in men. Overall, only 1.4% of incident cancers were attributable to overweight and obesity. The ASR of cancers attributable to overweight and obesity was 2.0 per 100,000. Postmenopausal breast cancer was the most common cancer attributable to overweight and obesity (*n* = 25; ASR 1.2 per 100,000).

**Conclusion:** Our results suggest that a small proportion of incident cancer cases in Nigeria are potentially preventable by maintaining normal body weight. The burden of cancer attributed to overweight and obesity in Nigeria is relatively small, but it may increase in future.

## Introduction

The incidence of overweight (body-mass index [BMI] ≥ 25 kg/m^2^–<30 kg/m^2^) and obesity (BMI ≥ 30 kg/m^2^) is rising globally. In 2015, an estimated 603.7 million adults were overweight and obese worldwide (1). Overweight and obesity alone contributed to 4.0 million deaths and 120 million disability-adjusted life-years in 2015, representing 7.1 and 4.9%, respectively, of death from any cause among adults globally (1). Globally, the prevalence of obesity has increased during the past three decades. However, both the trend and magnitude of BMI and BMI-related disease burden vary widely across countries (1).

Overweight and obesity are known risk factors for chronic diseases including certain cancers, cardiovascular and metabolic diseases (2–5). In 2012, it was estimated that approximately 481,000 or 3·6% of all new cancer cases in adults, aged 30 years and older after a 10-year lag period, were attributable to overweight and obesity worldwide (6). The burden of cancers attributable to overweight and obesity was higher in high-income countries (HIC) with population attributable fraction (PAF) of 5·3% compared to PAF of 1·6% in middle-income countries (MIC) and 1·0% in low-income countries (LIC) (6). Over the past few decades, the prevalence of overweight and obesity has increased markedly in Nigeria. Data from the Global Burden of Diseases (GBD) in 2015 reported an increase in the prevalence of overweight and obesity in Nigeria from 20% in 1980 to 74% in 2015 (1). In 2011, we found that the prevalence of overweight and obesity among urbanized adult Nigerians was 64%, with a higher prevalence in women (74%) than in men (57%) (5).

The International Agency for Research on Cancer (IARC) and the World Cancer Research Fund (WCRF) identified esophageal, colon, rectal, kidney, pancreatic, gallbladder (women only), postmenopausal breast, endometrial, and ovarian cancers as overweight and obesity associated cancers (7–13). There are country-specific estimates of cancers attributable to overweight and obesity for the United States of America (6.0%), United Kingdom (5.5%), Canada (5.1%), Australia (3.4%), and China (0.7%) (14–18). However, there are no country-specific estimates for sub-Saharan African countries including Nigeria, where the prevalence of overweight and obesity is increasing (19) and two-thirds of the urbanized adults are either overweight or obese (5). Given the high prevalence of adults with high BMI in Nigeria, estimating the proportion of cancers attributable to overweight and obesity would be informative to research programs and policy makers.

In this study, we used data from population-based cancer registries (PBCRs) to evaluate the incidence and proportion of cancers attributable to overweight and obesity from 2012 to 2014 in Nigeria.

## Methods

### Data Sources

We retrieved data on cancer incidence from Abuja and Enugu PBCRs in Nigeria, from 2012 to 2014. The details of the registries have been described elsewhere (20, 21). The Abuja Cancer Registry (ABCR) began in 2005 as a hospital-based cancer registry (HBCR). In 2009, the Nigerian National Systems of Cancer Registries (NSCR) provided the logistic support for the registry's upgrade to a PBCR. ABCR has a catchment area that covers the entire Federal Capital Territory with a population of 1,406,239 people, according to the national population census (22). The Enugu Cancer Registry (ECR) began in 1988 as a HBCR and was developed into a PBCR by the NSCR in 2012. It is domiciled in the Oncology Department of the University of Nigeria Teaching Hospital Ituku Ozalla, Enugu, Nigeria. ECR covers an area around the greater Enugu city metropolis with a population of 1,103,153 people (22). ABCR and ECR utilize the International Classification of Diseases for Oncology, 3rd Edition (ICD-O3) for coding and classification of cancers. ABCR uses CanReg4, while the ECR uses CanReg5 software for storing and processing data. Approval for cancer registration activities and research was obtained from the National Health and Research Ethics Committee of Nigeria (NHREC).

### Data Handling and Statistical Analysis

We obtained de-identified data in accordance with international best practices from both cancer registries. We cleaned the data to remove duplicates and carried out quality control checks using the in-built internal consistency checks in the IARC CanReg 5 software. We generated crude incidence and age-standardized incidence rates (ASR) using the direct method based on WHO World Standard Population. We extracted data on the cancers that have been shown to be associated with overweight and obesity. These cancers and their International Classification of Diseases for Oncology (ICD-O) codes are esophagus (C15), colon (C18), rectum (C19–20), kidney (C64), pancreas (C25), gallbladder (C23–24), postmenopausal breast (C50), endometrium (C54), and ovary (C56). Because there were no previous studies on cancers associated with overweight and obesity in Nigeria, we were unable to estimate the PAF for the study population. Thus, we used the PAF for overweight and obesity for SSA, derived in the Global burden of cancer attributable to high body-mass index by Arnold et al. (6), where PAF was estimated as the proportional reduction in population disease that would occur if exposure to a risk factor were reduced to an alternative ideal exposure scenario, as described by the WHO (23). We calculated the numbers of cancer cases attributable to overweight and obesity (ACalc) for each sex by multiplying the PAF of each cancer site with the overall numbers of cancer cases reported per cancer site (24). We carried out sensitivity analyses for cancers attributable to overweight and obesity, using the GLOBOCAN 2012 database. GLOBOCAN is a data source developed by IARC and provides estimates on cancer incidence, mortality, and prevalence for 184 countries of the world including Nigeria (25).

## Results

**Figure 1:**
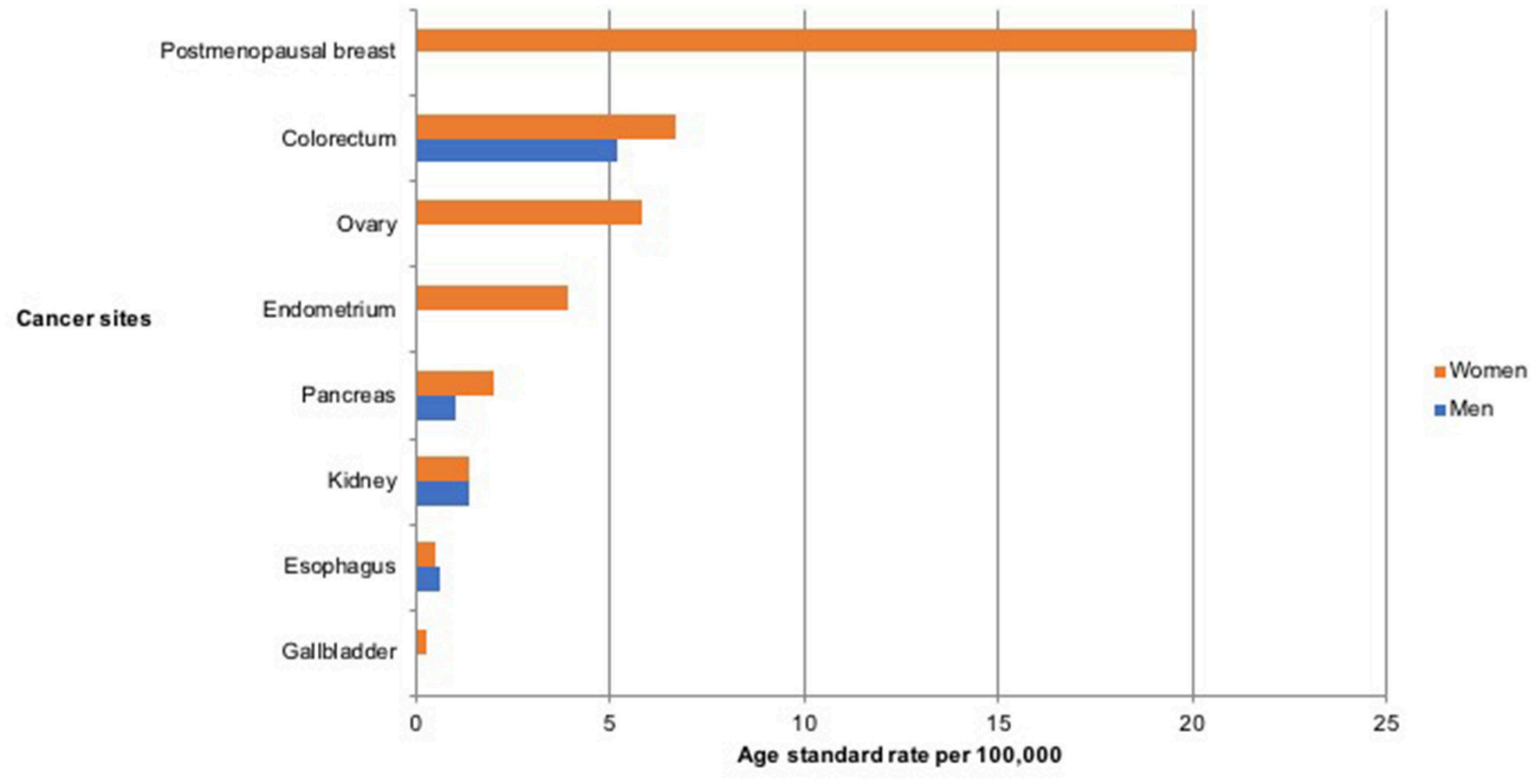
Age standardized incidence rate per 100,000 of cancers associated with overweight and obesity in Nigeria from 2012 to 2014.

A total of 4,336 new cancer cases were reported by the PBCRs within the study period, 2012–2014 (ASR 113.9 per 100,000). Among these, 2,709 (62.5%, ASR 145.9 per 100,000) were reported in women and 1,627 (37.5%, ASR 82.0 per 100,000) in men. Of the total cancers in both sexes, 21.0% (*n* = 907) were associated with overweight and obesity, with 81% (n=734) of these occurring in women and 19% (*n* = 173) in men (Figure 1). The ASR for overweight and obesity associated cancer was 24.5 per 100,000 in both sexes, 40.7 per 100,000 in women and 8.2 per 100,000 in men (Table 1).

**Table 1:** Population attributable fraction (PAF) and estimated numbers of cancers attributable to overweight and obesity in Nigeria from 2012 to 2014.

**Cancer site**	**ICD-O3 code**	**No. of cancer cases**	**% of total cancers**	**ASR of associated cancers**	**PAF %**	**Attributable cancers**	**ASR of attributable cancers**
**WOMEN**							
Postmenopausal breast	C50	412	15.2	20.1	6.1	25	1.2
Colorectum	C18–20	97	3.7	6.7	7.0	3	0.3
Corpus Uteri (Endometrium)	C54	44	1.6	3.9	24.8	11	1.0
Esophagus	C15	7	0.3	0.5	27.3	2	0.1
Gall bladder	C23-24	4	0.1	0.3	20.4	1	0.1
Kidney	C64	29	1.1	1.4	11.1	3	0.1
Ovary	C56	114	4.2	5.8	2.3	3	0.2
Pancreas	C25	27	1.0	2.0	5.6	2	0.1
Total cancers		734	27.1	40.7		50 (1.8%)	3.1
**MEN**							
Colon	C18–20	107	6.6	5.2	7.6	4	0.2
Esophagus	C15	12	0.7	0.6	15.5	2	0.1
Kidney	C64	35	2.2	1.4	5.9	2	0.1
Pancreas	C25	19	1.2	1.0	4.3	1	0.1
Total cancers		173	10.6	8.2		9 (0.6%)	0.4
**CANCERS IN BOTH SEXES**							
Colorectum	C18–20	204	4.7	6.0	7.3	7	0.3
Esophagus	C15	19	0.4	0.6	21.4	4	0.1
Kidney	C64	64	1.5	1.4	8.5	5	0.1
Pancreas	C25	46	1.1	1.5	5.0	3	0.1
Total cancers		907	21.0	24.5		59 (1.4%)	2.0

Some 1.4% (59 of 4,336) of the incident cancers in this population were attributable to overweight and obesity giving an ASR 2.0 per 100,000. Of these overweight and obesity attributable cancers, 84.7% (50 of 59) occurred in women giving an ASR of 3.1 per 100,000 while 15.3% (9 of 59) occurred in men giving an ASR 0.4 per 100,000.

### Postmenopausal Breast Cancer

Postmenopausal breast cancer was the commonest cancer associated with overweight and obesity in our study. The two registries reported 412 postmenopausal breast cancer cases from 2012 to 2014 (ASR 20.1 per 100,000). Of these, 6.1% (25 of 412) were attributable to overweight and obesity yielding an ASR 1.2 per 100,000 (6). Postmenopausal breast cancer was the most common cancer attributable to overweight and obesity in the study population.

### Colorectal Cancer

There were 204 cases of colorectal cancers reported by the two registries during the study period with an ASR of 6.0 per 100,000. About half (47.5%, *n* = 97, ASR 6.7 per 100,000) of this was in women and 52.5% (*n* = 107, ASR 5.2 per 100,000) was in men. Some 3.4% (7 of 204, ASR 0.3 per 100,000) of these cancers were attributable to overweight and obesity based on a PAF estimate of 7.0% in women and 7.6% in men (6). The ASR for colorectal cancers attributable to overweight and obesity was 0.3 per 100,000 in women and 0.2 per 100,000 in men.

### Ovarian Cancer

Ovarian cancer was the second most common cancer associated with overweight and obesity in women after postmenopausal breast cancer. There were 114 ovarian cancers reported by the registries (ASR 5.8 per 100,000). Of these, only 2.6% (*n* = 3, ASR 0.2 per 100,000) were attributable to overweight and obesity, using a PAF estimate of 2.3% (6).

### Kidney Cancer

There were 64 kidney cancer cases reported by the registries (ASR 1.4 per 100,000), with 45.3% (*n* = 29, ASR 1.4 per 100,000) in women and 54.7% (*n* = 35, ASR 1.4 per 100,000) in men. With a PAF estimate of 11.1% in men and 5.9% in women (6), we computed that 7.8% (*n* = 5, ASR 0.1 per 100,000) kidney cancer cases were attributable to overweight and obesity. The ASR for cases attributable to overweight and obesity was same in women and men, respectively (ASR 0.1 per 100,000).

### Pancreatic Cancer

Some 46 pancreatic cancer cases were reported within the study period (ASR 1.5 per 100,000), of which 58.7% (*n* = 27, ASR 2.0 per 100,000) were seen in women and 41.3% (*n* = 19, ASR 1.0 per 100,000) were seen in men. Using a PAF estimate of 5.6% in women and 4.3% in men (6), we estimated that 6.5% (*n* = 3, ASR 0.1 per 100,000) cases were attributable to overweight and obesity. The ASR for pancreatic cancer cases attributable to overweight and obesity was 0.1 per 100,000 in each of the sexes.

### Endometrial Cancer

Some 44 endometrial cancer cases were reported in women within the study period (ASR 3.9 per 100,000). Of these, we estimated that 25.0% (11 of 44, ASR 1.0 per 100,000) cases were attributable to overweight and obesity, using a PAF estimate of 24.8% (6).

### Esophageal Cancer

We found 19 esophageal cancer cases (ASR 0.6 per 100,000), with 36.8% (*n* = 7, ASR 0.5 per 100,000) in women and 63.2% (*n* = 12, ASR 0.6 per 100,000) in men. We computed that 21.1% (4 of 19, ASR 0.1 per 100,000) esophageal cancer cases were attributable to overweight and obesity, using a PAF estimate of 27.3% in women and 15.5% in men (6). The ASR for esophageal cancers attributable to overweight and obesity in women was 0.1 per 100,000 in both sexes.

### Gall Bladder Cancer (Women Only)

The registries reported 4 gall bladder cases in women (ASR 0.3 per 100,000). Of these, 25.0% (*n* = 1, ASR 0.3 per 100,000) was attributable to overweight and obesity using a PAF estimate of 27.3% (6).

### Premenopausal Breast Cancer

There were 805 cases of premenopausal breast cancers reported in women by the PBCRs from 2012 to 2014. The ASR for premenopausal breast cancer was 30.0 per 100,000. To determine how much more premenopausal breast cancer there may have been if the women were not overweight or obese, we calculated the association of overweight and obesity, and the reduced risk of premenopausal breast cancer using a relative risk of 0.95 from meta-analyses (26). We estimated that 847 premenopausal breast cancer cases (ASR 32.0 per 100,000), would have occurred if the premenopausal women were not overweight or obese.

### Sensitivity Analyses

GLOBOCAN estimated that 102,079 cancer cases occurred in Nigeria in 2012, 64,709 (63.3%) in women and 37,370 (36.6%) in men. Of these, some 22.1% (*n* = 22,477, ASR 21.5 per 100,000) were associated with overweight and obesity, 83.7% (18,820 of 22,477) in women and 16.3% (3,657 of 22,567) in men (Table 2). Of the overweight and obesity associated cancers reported by GLOBOCAN, 1.7% (1,707 of 102,079, ASR 2.0 per 100,000) were attributable to overweight and obesity, with 1,457 (2.9%, ASR 2.9 per 100,000) in women and 250 (0.6%, ASR 0.6 per 100,000) in men (Table 2).

**Table 2:** Sensitivity analyses of cancers attributable to overweight and obesity in Nigeria from GLOBOCAN 2012 database.

**Cancer site**	**ICD-O3 code**	**No. of cancer cases**	**% of total cancers**	**ASR of associated cancers**	**PAF %**	**Attributable cancers**	**ASR of attributable cancers**
**WOMEN**							
Postmenopausal breast	C50	12,036	18.6	22.3	6.1	734	1.4
Colorectum	C18–20	2,008	3.1	4.0	7.0	140	0.3
Corpus Uteri (Endometrium)	C54	1,563	2.4	3.4	24.8	388	0.8
Esophagus	C15	141	0.2	0.3	27.3	38	0.1
Gall bladder	C23–24	88	0.1	0.2	20.4	18	0.0
Kidney	C64	523	0.8	0.7	11.1	58	0.1
Ovary	C56	1,723	2.7	3.1	2.3	40	0.1
Pancreas	C25	738	1.1	1.5	5.6	41	0.1
Total cancers		18,820	29.1	35.5		1,457 (2.3%)	2.9
**MEN**							
Colorectum	C18–20	2,164	5.8	4.5	7.6	164	0.3
Esophagus	C15	145	0.4	0.3	15.5	22	0.0
Kidney	C64	413	1.1	0.6	5.9	24	0.0
Pancreas	C25	935	2.5	2.2	4.3	40	0.1
Total cancers in men		3,657	10.0	7.6		250 (0.6%)	0.4
**CANCERS IN BOTH SEXES**							
Colorectum	C18–20	4,172	4.1	4.3	7.3	304	0.3
Esophagus	C15	286	0.3	0.3	21.4	60	0.1
Kidney	C64	936	1.0	0.7	8.5	82	0.1
Pancreas	C25	1,673	1.6	2.0	5.0	81	0.1
Total cancers		22,477	22.1	21.5		1,707 (1.7%)	2.0

Postmenopausal breast cancer was the commonest cancer attributable to overweight and obesity in Nigeria as evident from the two independent data sources. This cancer represents 1.2% (25 of 4,336) in data from Nigerian PBCRs and 1.4% (734 of 102,079) in data from GLOBOCAN 2012.

## Discussion

This study showed that 1.4% of incident cancer cases in Nigeria were attributable to overweight and obesity. In our study, a higher proportion of cancers were attributable to overweight and obesity in women (1.8%), than men (0.6%). The most common cancers attributable to overweight and obesity in women were postmenopausal breast cancers (ASR 1.2 per 100,000) and endometrial cancers (ASR 1.0 per 100,000). These cancers contributed about three-quarters (72.0%) of attributable cancers in women and more than half (61.0%) of attributable cancers in both sexes. Colon, kidney and esophageal cancers with ASR 0.1 per 100,000 each, were the most common cancers attributable to overweight and obesity in men. These cancers accounted for 77.8% of total attributable cancers in men.

Our findings of incident cancer cases attributable to overweight and obesity are similar to findings from other developing countries, but lower than global estimates (6) and findings from developed countries such as United States (6.0%), United Kingdom (5.5%), and Australia (3.4%), where there is a higher prevalence of overweight and obesity (14, 15, 17).

In Nigeria, the burden of overweight and obesity is high and increasing (1, 5). As the Nigerian economy continues to improve and food becomes more widely available and affordable, individuals' dietary calorie intake will increase and contribute to the rising prevalence of overweight and obesity (5). Other factors shown to be associated with overweight and obesity among Nigerian adults include age, female gender, marital status, high socio-economic status, urban residence, and sedentary lifestyle (5, 27, 28). The proportion of people who engage in recreational physical activity in Nigeria is low. In a previous study, we found that up to two-thirds of our study population in urban Nigeria, did not engage in significant recreational physical activity (29). Our findings from this study showed that the burden of cancers attributable to overweight and obesity is higher in women, and these are women who are also likely to be obese and less likely to engage in recreational physical activities in this population.

Postmenopausal breast cancer was the most common cancer attributable to overweight and obesity in our study. This is similar to findings from other countries in sub-Saharan Africa (SSA) (6). Postmenopausal breast cancer contributed to half of cancers attributable to overweight and obesity in women and 42.4% of all attributable cancers in both sexes. The association between obesity and the risk of breast cancer is modified by menopausal status, with obesity directly associated with increased risk of breast cancer in postmenopausal women and reduced risk in premenopausal women (30). The increased risk of postmenopausal breast cancer in obese women has been suggested to be due to the high estrogen production, resulting in increased endogenous exposure to estrogen and increased risk of breast cancer (31–33). Conversely, some studies have shown that women who were overweight or obese during premenopausal ages may have a reduced risk of breast cancer compared to women of normal body weight (26, 34, 35). A potential biological mechanism for this inverse association is that among premenopausal women, overweight and obesity is associated with anovulation and lower levels of circulating estrogen levels (36).This is in contrast to overweight and obese postmenopausal women that have elevated circulating estrogen levels (26).

Colorectal cancer was the second most common cancer associated with overweight and obesity in both sexes in Nigeria. The incidence of colorectal cancers attributable to overweight and obesity in our study was higher in females than males, similar to findings from SSA countries (6). The rising incidence of colorectal cancers is associated with excessive high-fat and low-fiber diet, consumption of refined products, lack of physical activity, and obesity (31, 32). The relationship between obesity and colorectal cancer development can be attributed to metabolic syndrome and expression of various adipokines (leptin and adiponectin) which drive colorectal cancer development (33, 37, 38).

Endometrial cancer is the second most common cancer attributable to overweight and obesity in Nigerian women after breast cancer. Approximately 22% of endometrial cancer cases in Nigeria were attributable to overweight and obesity in our study. This estimate was lower than that reported in the US (34%) and US (47%). The higher prevalence of endometrial cancer attributable to overweight and obesity in developed countries may be due to the high prevalence of overweight and obesity, increased cancer awareness, opportunities for wellness checks and more resources for diagnosis in those countries. The proportion of endometrial cancers attributable to overweight and obesity in Nigeria may increase, as the country continues to develop. Other risk factors for endometrial cancers include older age, early menarche, late menopause, family history of endometrial cancer, and long-term use of estrogen for hormone replacement therapy (39). Obesity is associated with pro-inflammatory adipokines stimulation, estrogen and progesterone imbalance as well as dysregulation of insulin and insulin-like growth factor activity, which collectively contribute to endometrial proliferation and carcinogenesis (40). Although lifestyle and diet modification have been considered to reduce the risk of developing endometrial cancer, these findings were inconclusive (41–43).

Gallbladder cancer is uncommon globally and in the Nigerian population, except in areas with high prevalence of *Clonorchis sinensis* which is associated with cholangiocarcinoma such as the Koreas. The incidence of gallbladder cancer in Nigeria (ASR 0.2 per 100,000) is similar to findings from other SSA countries, but lower than the findings from Europe and American regions (44). This may be due to the fact that gallbladder cancer is underreported in SSA countries (45, 46). Gallstones, alcohol consumption, smoking, diabetes mellitus, genetic susceptibility, and obesity are major risk factors for gall bladder cancer (47–51). The risk of overweight associated gall bladder cancers has been reported to be more significant in women than men, a finding that is consistent with our study (6, 52, 53). Studies has shown that obesity increases the risk of gall bladder cancer by altering lipid and endogenous hormone metabolism, promoting formation of gallstones, and elevating blood glucose level (50, 54).

Ovarian cancer is the third most common gynecological cancer among Nigerian women after breast and cervical cancer (55). The incidence of ovarian cancer attributable to overweight and obesity in our study is consistent with findings from other SSA countries (6). The lack of knowledge of the premalignant stage of ovarian cancer and non-availability of a screening tool makes early diagnosis of this cancer difficult (56). This results in the late presentation and poor prognosis associated with ovarian cancer in Nigeria (57, 58). Studies suggest that the binding of leptin to its receptor (OB-Rb), activates the signaling pathways for ovarian cancer progression, particularly in obese women, resulting in a poor survival rate (59, 60).

Kidney cancer is responsible for 2.4% of the total cancer burden worldwide and 1.0% of total cancers reported in Nigeria in 2012 (44). Result from prospective studies showed that overweight and obese individuals at baseline were found to have an elevated subsequent risk of kidney cancers in a dose-response manner (61–63). In our study, approximately 8.5% of incident kidney cancer cases were attributable to overweight and obesity, which is lower than findings from developed countries (6). The higher incidence of kidney cancer in developed countries can be attributable to early diagnosis, availability of better diagnostic tools and the high prevalence of risk factors which include smoking, obesity, physical inactivity, high blood pressure and occupational exposure to trichloroethylene, cadmium and asbestos (64, 65).

The incidence of pancreatic cancer varies greatly across different populations and regions of the world with higher rates in developed countries, compared to developing countries (6). Like many other developing countries, pancreatic cancer diagnosis in Nigeria is associated with late presentation resulting in poor survival (66). In our study, pancreatic cancer accounted for 5.1% of cancers attributable to overweight and obesity. Other risk factors for pancreatic cancer include cigarette smoking, positive family history and genetics, heavy alcohol use, diabetes mellitus, dietary factors and physical inactivity (67, 68).

Our study has several limitations. We included data from two out of six PBCRs in Nigeria because the other PBCRs were newly established and did not have data covering the time period reviewed. Nonetheless, the two PBCRs used in our study represent distinct regions of Nigeria (North-Central and South-East) with nationally representative socio-demographic characteristics. Given the dearth of data, we were unable to estimate Nigerian-specific PAF. Thus, we used the estimates from the GBD, which may not be a true representation of the Nigerian population. Also, a 10-year lag period between obesity and incident cancer was assumed in the study where our PAF was obtained. We could not account for time lived with overweight or obesity in our analyses. Lastly, we cannot rule out the possibility of the incompleteness of cancer data and under-reporting from the two PBCRs considered in this study.

However, to our knowledge, this study is the first to evaluate country-specific incidence of cancers attributable to overweight and obesity in Africa. Our findings on the incidence of cancers attributable to overweight and obesity in this study (1.4%, ASR 2.0 per 100,000) are further strengthened by the results of the sensitivity analysis, which were similar to the results reported in GLOBOCAN 2012 for Nigeria (1.7%, ASR 2.0 per 100,000).

## Conclusion

Our results suggest that 1.4–1.7% of incident cancer cases in Nigeria are potentially preventable by maintaining normal body weight. The burden of cancer attributed to overweight and obesity is still relatively small in Nigeria, but it is likely to increase in future. Findings from our study may help guide decision making on control and prevention of overweight and obesity, which may reduce the burden of these cancers in future.

## Author Contributions

MO and SA contributed to data collection, data analyses, and drafting the manuscript. TO, FI, TIO, EE, RH, and EJ-A contributed to the data collection and data quality. SA guided all aspects of the manuscript and provided critical revisions. All authors contributed to the manuscript and approved the final version.

### Conflict of Interest Statement

The authors declare that the research was conducted in the absence of any commercial or financial relationships that could be construed as a potential conflict of interest. The reviewer EAO declared a past co-authorship with several of the authors MO, FI, TIO, EE, RH, EJ-A and SA to the handling editor.
